# Strategies to improve genomic predictions for 35 duck carcass traits in an F_2_ population

**DOI:** 10.1186/s40104-023-00875-8

**Published:** 2023-05-06

**Authors:** Wentao Cai, Jian Hu, Wenlei Fan, Yaxi Xu, Jing Tang, Ming Xie, Yunsheng Zhang, Zhanbao Guo, Zhengkui Zhou, Shuisheng Hou

**Affiliations:** 1 Institute of Animal Science, Chinese Academy of Agricultural Sciences, Beijing, 100193 China; 2 Shandong New Hope Liuhe Group Co., Ltd., Qingdao, 266108 China; 3 College of Animal Science and Technology, Qingdao Agricultural University, Qingdao, 266109 China; 4 College of Animal Science and Technology, Beijing University of Agriculture, Beijing, 102206 China

**Keywords:** Bayesian model, Carcass traits, Duck, Genome prediction, Genomic relationship matrix, Mark density

## Abstract

**Background:**

Carcass traits are crucial for broiler ducks, but carcass traits can only be measured postmortem. Genomic selection (GS) is an effective approach in animal breeding to improve selection and reduce costs. However, the performance of genomic prediction in duck carcass traits remains largely unknown.

**Results:**

In this study, we estimated the genetic parameters, performed GS using different models and marker densities, and compared the estimation performance between GS and conventional BLUP on 35 carcass traits in an F_2_ population of ducks. Most of the cut weight traits and intestine length traits were estimated to be high and moderate heritabilities, respectively, while the heritabilities of percentage slaughter traits were dynamic. The reliability of genome prediction using GBLUP increased by an average of 0.06 compared to the conventional BLUP method. The Permutation studies revealed that 50K markers had achieved ideal prediction reliability, while 3K markers still achieved 90.7% predictive capability would further reduce the cost for duck carcass traits. The genomic relationship matrix normalized by our true variance method instead of the widely used $\sum {2p}_{i}(1-{p}_{i})$ could achieve an increase in prediction reliability in most traits. We detected most of the bayesian models had a better performance, especially for BayesN. Compared to GBLUP, BayesN can further improve the predictive reliability with an average of 0.06 for duck carcass traits.

**Conclusion:**

This study demonstrates genomic selection for duck carcass traits is promising. The genomic prediction can be further improved by modifying the genomic relationship matrix using our proposed true variance method and several Bayesian models. Permutation study provides a theoretical basis for the fact that low-density arrays can be used to reduce genotype costs in duck genome selection.

**Supplementary Information:**

The online version contains supplementary material available at 10.1186/s40104-023-00875-8.

## Introduction

Ducks play a considerable role in the structure of the waterfowl meat market in Asia and some European countries. Duck is also the third largest meat consumption in China after pork and chicken. Duck cuts, such as breasts and legs, offer more options for diet-conscious consumers. Duck meat is generally regarded as flavorsome, rich in amino acids and polyunsaturated fatty acids, and relatively low in fat. Duck meat has a higher number of muscle fibers [1], lipid contents [2], lower water-holding capacity, and greater cooking loss [3] compared to chicken meat. Ducks’ other products, such as neck, liver, gizzard, and feet were also popular. As these products could be processed into different ready-to-eat meat products, such as roasted Pekin duck, Nanjing salted duck, and spicy duck neck.

Several efforts have been made to improve duck productive traits in duck breeding programs using pedigree and phenotypic information [4]. Although the selection for productive traits is feasible in ducks, measuring duck carcass traits increases costs and can recorded mainly after slaughter. Furthermore, for these traits that cannot be measured in vivo, sib-testing is a routine method used in traditional selection, with only the between-family variation to be used, which limits the selection accuracy. For the above reasons, the current duck breeding programs do not pay much attention to the improvement of carcass composition traits [5].

Genomic selection (GS) is an effective approach in animal breeding to improve selection and reduce costs, which has been widely used in livestock [6], poultry [7] and aquatic animals [8]. Genomic prediction combines genotypic, phenotypic, and pedigree data to increase the accuracy of estimates of genetic merit and to decrease generation intervals [6]. To date, the use of genome prediction in ducks has rarely been investigated. The only one reporting duck genome prediction is about the meat quality traits of the Peking duck [9]. However, this study only focused on seven traits: the weight and percentage of abdominal fat, skin fat and breast muscle, as well as live weight. Genomic selection in many important carcass traits (like carcass weight, eviscerated weight, dressing rate etc.) remains uncovered. Moreover, compared to the best linear unbiased prediction (BLUP), the advantage of genome prediction had not been reflected due to their small sample size [9, 10].

In ducks, a commercial single nucleotide polymorphism (SNP) array has not been developed, the only way to get genotype information is from whole or reduced-representation genome sequencing. A lack of low-cost SNP arrays would increase the breed cost and delay the application of genomic selection. Marker density is an important factor affecting the accuracy of genome prediction and breed cost [11]. Although high-density markers can improve prediction accuracy, when the marker density reached a certain degree, there will be no further meaningful increase in prediction accuracy [12, 13]. On the contrary, the breeding cost will be dramatically increased. The optimal marker density for duck GS, such as the density reaching a plateau, remains obscure, since the efficient SNP number could reduce the dimensionality of the GS model and breeding cost.

The choice of statistical models also has a noticeable impact on the prediction accuracy of GS [14, 15]. The genomic best linear unbiased prediction (GBLUP) method has been widely used in routine genomic evaluation because it is easier to implement and less computationally demanding [16]. As the construction of the genomic relationship matrix (GRM) immediately affects the GBLUP model, many efforts have been made to modify GRM, which uses unequal weights for all SNPs [17–19]. Compared to the GBLUP module, Bayesian models have the advantage of modeling the distribution of marker effects [20], which helps increase the GS accuracy in various studies [21, 22]. However, the advantages of the modified GBLUP and multiple Bayesian methods have not been evaluated for carcass traits in ducks.

Duck meat production is based mainly on commercial crossbreeds of different Pekin strains. Hence, the objectives of this study were: (1) to calculate genetic parameters of duck carcass traits, (2) to estimate reliability gains from using genomic evaluations instead of traditional BLUP evaluations, (3) to document how the density of markers affect predictive ability of GS, (4) to propose the best strategies to improve genomic predictions for duck carcass traits.

## Materials and methods

### Experimental population, phenotype, and genotype data

The phenotypes, pedigrees, and genotypes of ducks were conducted in an experimental cross-population of Pecking duck × mallard. Phenotypes for 35 carcass composition traits were measured in 988 animals with age of 8 weeks. Table 1 describes the number of animals with observations with mean and standard deviation (SD) for each trait. The 914 ducks were selected for sequencing on an Illumina HiSeq X Ten with an average × 5 coverage. The detailed information of sequencing data had been described in our previous study [23]. The 150-bp paired-end clean reads were mapped to the Pekin duck reference genome (GCA_003850225.1) with BWA (v0.7.17) [24]. The alignment quality was improved by Picard (v2.24.1) [25]. The SNP calling was conducted by GATK HaplotypeCaller module (v3.5) [26]. The SNPs were removed according to the following criteria: (a) non-autosomal variants, (b) minor allele frequency (MAF) < 0.05, (c) call rate < 0.9, (d) individuals missing more than 10% of genotypes were removed. Quality control of genotype data was conducted using PLINK (v1.90) [27]. After filtering, 1,037,662 SNPs for 914 individuals were retained in the dataset.

**Table 1:** Summary statistics and heritabilities (*h*^2^) estimation for the 35 phenotypic traits

Traits	Mean	SD	Records	Heritability	*h*^2^ SE
Weight traits, g					
Carcass weight	1,632.46	234.86	987	0.54	0.08
Eviscerated weight	1,491.01	214.17	984	0.51	0.07
Breast muscle weight	86.15	15.73	988	0.44	0.07
Leg muscle weight	89.74	14.02	987	0.46	0.07
Skin and subcutaneous fat weight	376.73	89.79	988	0.60	0.08
Abdominal fat weight	32.52	12.68	975	0.63	0.08
Skeleton weight	597.42	79.47	988	0.43	0.07
Head weight	77.19	9.66	988	0.47	0.07
Neck weight	88.11	13.02	987	0.31	0.06
Swing weight	72.69	9.57	965	0.43	0.07
Heart weight	11.70	1.81	976	0.41	0.07
Gizzard weight	52.37	9.91	987	0.41	0.07
Liver weight	35.84	7.06	973	0.32	0.06
Feet weight	41.27	6.33	988	0.51	0.07
Length traits, cm					
Neck length	22.54	1.51	983	0.17	0.05
Total intestine length	144.57	14.91	966	0.36	0.07
Duodenum length	26.67	2.84	967	0.21	0.06
Jejunum length	117.90	13.35	966	0.35	0.07
Ileum length	14.63	1.54	966	0.37	0.07
Shank length	5.19	0.26	942	0.16	0.05
Percentage traits, %					
Dressed percentage	85.80	2.77	984	0.08	0.05
Eviscerated carcass	78.39	2.97	984	0.10	0.05
Lean meat	23.64	1.59	984	0.37	0.07
Breast muscle	11.55	1.45	984	0.38	0.07
Leg muscle	12.09	1.33	984	0.30	0.07
Skin & subcutaneous fat	21.29	2.38	984	0.55	0.08
Abdominal fat	2.10	0.67	971	0.56	0.08
Skeleton	35.33	1.97	986	0.28	0.06
Head	5.21	0.48	987	0.28	0.06
Neck	5.93	0.56	986	0.47	0.07
Swing	4.90	0.38	961	0.27	0.06
Heart	0.72	0.09	975	0.25	0.06
Gizzard	3.23	0.54	986	0.45	0.07
Liver	2.20	0.33	972	0.09	0.05
Feet	2.78	0.33	987	0.24	0.06

The percentage was abbreviated as % in trait name. The standard deviation of phenotype was denoted by SD. The standard error of *h*^2^ was denoted by SE

### The estimation of genetic parameters with the BLUP model

The animal model is

$$\begin{array}{c}{\varvec{y}} =\boldsymbol{ }{\varvec{X}}{\varvec{\beta}}+ {\varvec{Z}}{\varvec{a}} +{\varvec{e}},\\ {\varvec{V}}{\varvec{a}}{\varvec{r}}\boldsymbol{ }\left[\begin{array}{c}{\varvec{a}}\\ {\varvec{e}}\end{array}\right]=\left[\begin{array}{cc}{\varvec{A}}{{\varvec{\sigma}}}_{{\varvec{a}}}^{2}& 0\\ 0& {\varvec{I}}{{\varvec{\sigma}}}_{{\varvec{e}}}^{2}\end{array}\right],\end{array}$$

where ***y*** − the vector of phenotype value; ***β*** − the vector of fixed effects, including sex, reciprocal crosses and feed room; ***a*** − the vector of additive genetic effects and assumed that ***a***∼N(0, ***A***${\sigma }_{a}^{2}$), in which ***A*** − the matrix of an additive genetic relationship constructed based on the pedigree; ${\sigma }_{a}^{2}$ − the additive genetic variance; ***X*** − incidence matrix for fixed effects; ***Z*** − incidence matrix to allocate phenotypic observations to individuals; ***e*** − random residual effects, ${\upsigma }_{e}^{2}$ is error variance. The narrow sense heritability (${h}^{2}$) was calculated by ${\sigma }_{a}^{2}$*/*$({\sigma }_{a}^{2}+{\sigma }_{e}^{2})$. According to the definition of a previous study [28], we defined that the moderate heritability ranged from 0.20 to 0.40, and high when it was greater than or equal to 0.40, while the low heritability should be less than or equal to 0.2. The estimation of the variance components, genetic correlation and breeding values was performed by restricted maximum likelihood (REML) analysis implemented in the ASReml-R (V4.2) [29]. The correlation coefficient values were interpreted as follows [30]: 0.0–0.2 little; 0.2–0.4 weak; 0.4–0.7 moderate; 0.7–1.0 strong.

### Genome prediction with the GBLUP model

The linear mixed models are formulated as

$${\varvec{y}} = {\varvec{X}}{\varvec{\beta}}+ {\varvec{Z}}{\varvec{g}} + {\varvec{e}}$$

where ***y***, ***X***, ***β***, and ***e*** are the same as in the BLUP model, while ***g*** − vectors of additive genetic values. GBLUP was calculated using the genomic marker information provided by the SNPs. The genomic relationship matrix was calculated by VanRaden's method [16], the formula was listed as follows:

$${\varvec{G}}=\frac{{\varvec{Z}}{\varvec{Z}}\boldsymbol{^{\prime}}}{2\sum {p}_{i}(1-{p}_{i})}$$

where ***Z*** − the SNP markers’ incidence matrix, and it is the genotype matrix (***M***, 0 1 2) minus the mean of marker across individuals 2${p}_{i}$, ***Z*** = ***M*** − 2${p}_{i}$, where ${p}_{i}$ is the minor allele frequency (MAF) at each SNP.

### Building GRM using different methods

The GRM construction method proposed by VanRaden [16] was widely used in the genome prediction of animals and plants, which has been mentioned above. This method assumed that each marker has the same variance of genotype across individuals, then the ***ZZ'*** should be divided by $\sum {2p}_{i}(1-{p}_{i})$, meaning the markers’ weighting was equal.

The second method was commonly used by human genetics studies, such as Yang et al.’s GCTA [17, 31], as follows:

$${\varvec{G}} = {\varvec{Z}}{\varvec{D}}{\varvec{Z}}\boldsymbol{^{\prime}}$$

***Z*** is mentioned above, ***D*** − a diagonal matrix with ${D}_{ii}$,

$${D}_{ii}=\frac{1}{m[2{p}_{i}\left(1-{p}_{i}\right)]}$$

where *m* is the number of SNP markers. This method assumed that each marker has a different variance of genotype across individuals. Each marker should be scaled by itself variance $2{p}_{i}\left(1-{p}_{i}\right)$, meaning the markers’ weighting was different.

Both of the above two methods used the $2{p}_{i}\left(1-{p}_{i}\right)$ as the variance of each SNP genotype. The reason is that they assumed that the markers obey the Hardy–Weinberg principle. Then, for the $i$^th^ SNP with two alleles, one allele with frequencies ${p}_{i}$, we could know that the genotype (0, 1, 2) frequencies under random mating were

$$\begin{array}{c}{f\left(0\right)=\left(1-{p}_{i}\right)}^{2},\\ f\left(1\right)=2{p}_{i}\left(1-{p}_{i}\right),\\ f\left(2\right)={p}_{i}^{2}.\end{array}$$

The expectation ${E}_{i}$ and variance ${V}_{i}$ under the Hardy–Weinberg principle would be:

$$\begin{array}{c}E_i=1\times2p_i\left(1-p_i\right)+2\times p_i^2=2p_i,\\V_i=\left(0-E_i\right)^2\times f\left(0\right)+\left(1-E_i\right)^2\times f\left(1\right)+\left(2-E_i\right)^2\times f\left(2\right)=2p_i\left(1-p_i\right).\end{array}$$

However, in most cases, the SNPs could be influenced by artificial selection, inbreed, mutation, genetic drift, etc., which were not fully satisfied for the Hardy–Weinberg principle. Using ${p}_{i}\left(1-{p}_{i}\right)$ as variance would bring a bias. Here we used each marker's true variance ${{Var}_{i}}^{*}$ instead of $2{p}_{i}\left(1-{p}_{i}\right)$. The true variance ${{Var}_{i}}^{*}$ of each marker could be immediately calculated from the genotypes across individuals. Here, we assumed that the frequencies of $i$^th^ SNP genotypes 1 and 2 were ${p}_{1i}$ and ${p}_{2i}$, respectively. Then

$$\begin{array}{l}{f\left(0\right)}^{*}= 1-{p}_{1i}-{p}_{2i},\\{f\left(1\right)}^{*}= {p}_{1i},\\{f\left(2\right)}^{*}={p}_{2i},\end{array}$$

The true expectation ${{E}_{i}}^{*}$ and variance ${{Var}_{i}}^{*}$ would be:

$$\begin{array}{c}E_i^\ast=1\times p_{1i}+2\times p_{2i}=p_{1i}+2p_{2i}\\{Var}_i^\ast=\left(0-E_i^\ast\right)^2\times{f\left(0\right)}^\ast+\left(1-E_i^\ast\right)^2\times{f\left(1\right)}^\ast+\left(2-E_i^\ast\right)^2\times{f\left(2\right)}^\ast=p_{1i}+4p_{2i}-{{(p}_{1i}+{2p}_{2i})}^2.\end{array}$$

When the SNP marker across individuals obeys the Hardy–Weinberg principle, its MAF ${{p}_{i}=0.5p}_{1i}+{p}_{2i}$. Then ${{E}_{i}}^{*}$ and ${{Var}_{i}}^{*}$ were equal to ${E}_{i}$ and ${V}_{i}$, respectively.

Using ${p}_{1i}$ and ${p}_{2i}$ instead of MAF ${p}_{i}$, we could get the modified VanRaden’s formula:

$${\varvec{G}}=\frac{({\varvec{M}}-{p}_{1i}-2{p}_{2i})({\varvec{M}}-{p}_{1i}-2{p}_{2i})\mathrm{^{\prime}}}{\sum [{p}_{1i}+4{p}_{2i}-{{(p}_{1i}+{2p}_{2i})}^{2}]}.$$

The modified Yang et al.’s formula should be:

$${\varvec{G}}=\left({\varvec{M}}-{p}_{1i}-2{p}_{2i}\right){\varvec{D}}({\varvec{M}}-{p}_{1i}-2{p}_{2i}).$$

The markers’ diagonal ***D*** matrix should be:

$${D}_{ii}=\frac{1}{m[{p}_{1i}+4{p}_{2i}-{{(p}_{1i}+{2p}_{2i})}^{2}]}$$

Therefore, we generated four different GRM models used for estimating SNP heritability and genome prediction reliability: VanRaden’s model (Sum 2*p*(1 − *p*), SP), Yang’s model (Independent 2*p*(1 − *p*), IP), VanRaden’s model with true variance (Sum *Var*, SV) and Yang et al.’s model with true variance (Independent *Var*, IV).

### Bayesian models

The module is

$${\varvec{y}} = {\varvec{X}}{\varvec{\beta}}+ {\varvec{Z}}{\varvec{s}} + {\varvec{e}}$$

where ***y***, ***X***, ***β***, ***Z***, and ***e*** are the same as terms described in GBLUP model, and ${\varvec{s}}$− the sum of the vector of SNP effects derived from different assumed distributions. Here, we used five Bayesian models with different assumed distributions of SNP effects. BayesB assumes that most of the genetic markers have zero effect, which can be described as a mixture prior of a scaled *t*-distribution with probability π and a point mass at 0 with probability 1 − *π* [20]. BayesCπ assumes that SNP effects have a mixture prior of a normal distribution that has mean 0 and variance σ^2^ with probability π and null effect markers with probability 1 − *π* [32]. BayesN is the nested BayesCπ model, where the SNPs within a 0.2 Mb non-overlapping genomic region are collectively considered as a window. BayesS is similar to BayesCπ but the variance of SNP effects (for SNPs with non-zero effects) is related to MAF (${p}_{i}$) through a parameter *S* (${\sigma }_{i}^{2}={[2{p}_{i}\left(1-{p}_{i}\right)]}^{S}{\sigma }^{2}$) [33]. BayesR assumes that SNP effects follow a mixture of four normal distributions N(0,${\gamma }_{k}{\sigma }_{k}^{2}$), the ${\gamma }_{k}$ are 0, 0.01, 0.1 and 1 with probability ${\pi }_{1}$, ${\pi }_{2}$, ${\pi }_{3}$ and ${\pi }_{4}$, respectively, and ${\pi }_{1}+{\pi }_{2}+{\pi }_{3}{+ \pi }_{4}=1$ [34]. The unknown parameters and SNP effects of Bayesian models were obtained from a Gibbs scheme based on the Markov chain Monte Carlo (MCMC) iterations implemented in the GCTB (V2.01) software [33].

### Cross-validation and prediction reliability

The prediction reliability of the models was estimated based on a fivefold cross-validation. In fivefold cross-validation, the phenotypes of 20% of the animals were masked and then estimated using the phenotypes and genotypes of the remaining 80% of animals. The dataset of genotyped animals with phenotypes was randomly divided into five subsets, predicting one subset at a time and using the phenotypes of the remaining four subsets. Genomic prediction reliability was calculated as the Pearson correlation coefficient between adjusted phenotypic values and genomic predicted genetic values. The mean correlation value was used as the reliability for each trait.

### The permutation of marker densities

To evaluate the influence of marker density on the SNP heritability estimation and genome prediction, we randomly selected 0.5K, 1K, 3K, 5K, 10K, 50K, 100K and 500K from the original 1.04 million (M) markers. We built the genomic relationship matrix, and estimate SNP heritabilities and genomic breeding values using the GBLUP model for each selected marker. The prediction reliability of the models was estimated based on a fivefold cross-validation. We repeated this process 30 times to obtain stable results for each marker density. The predictive capability was equal to the current reliability divided by the best performance of reliability within the nine different density markers for a given trait.

## Results

### The genetic parameters

**Fig. 1:**
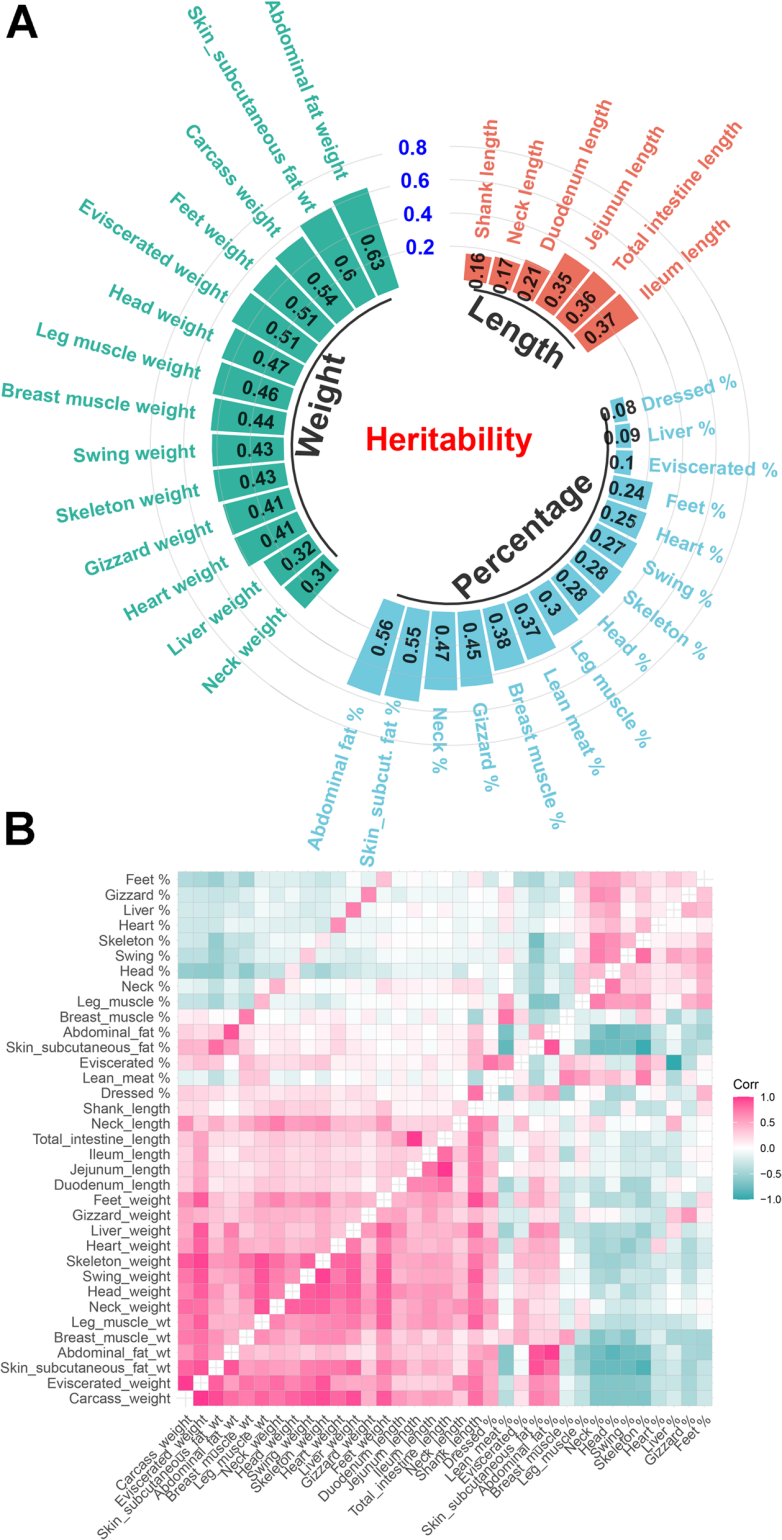
The genetic parameters of duck carcass traits. **A** The heritabilities of carcass traits, containing 14 weight traits, 6 length traits, and 15 percentage traits, were marked by green, orange, and blue color, respectively. **B** The genetic correlation (above the diagonal) and phenotype correlation (below the diagonal) between carcass traits. The color of each box represents a positive correlation (red) or a negative correlation (green)

The phenotype information of 35 traits was described in Table 1, which contained the mean and SD for each trait. The estimates of heritability based on the pedigree BLUP model were also presented in Table 1 and Fig. 1A. The heritability estimations were high for the abdominal fat weight (0.63), skin and subcutaneous fat weight (0.60), abdominal fat percentage (0.56), skin and subcutaneous fat percentage (0.55), carcass weight (0.54), eviscerated weight (0.51) and feet weight (0.51), while some traits had relatively low heritability, such as dressed rate (0.08), liver percentage (0.09), eviscerated percentage (0.10), shank length (0.16) and neck length (0.17). We detected both phenotypic and genetic relationships between the weight/length traits were usually positive. As expected, the weight traits were also positively correlated with their corresponding percentage traits (Fig. 1B and Table S1). The strongest correlations were observed between jejunum length and total intestine length (*r*_*g*_ = 0.998 ± 0.002, *r*_*p*_ = 0.976 ± 0.004), carcass weight and eviscerated weight (*r*_*g*_ = 0.994 ± 0.002, *r*_*p*_ = 0.976 ± 0.004). The negative correlation between some of the percentage traits was due to their competitive ratio on whole carcass. Interestingly, we found the gaps between genetic and phenotype correlation were considerable in several trait pairs (absolute value > 0.8), such as eviscerated percentage and liver (*r*_*g*_ = −0.976 ± 0.328, *r*_*p*_ = −0.089 ± 0.045), lean meat percentage and skeleton weight (*r*_*g*_ = 0.706 ± 0.127, r_p_ = −0.133 ± 0.071), and dressed rate and shank length (*r*_*g*_ = 0.774 ± 0.275, *r*_*p*_ = −0.026 ± 0.049).

### Genome prediction performance for carcass traits

**Fig. 2:**
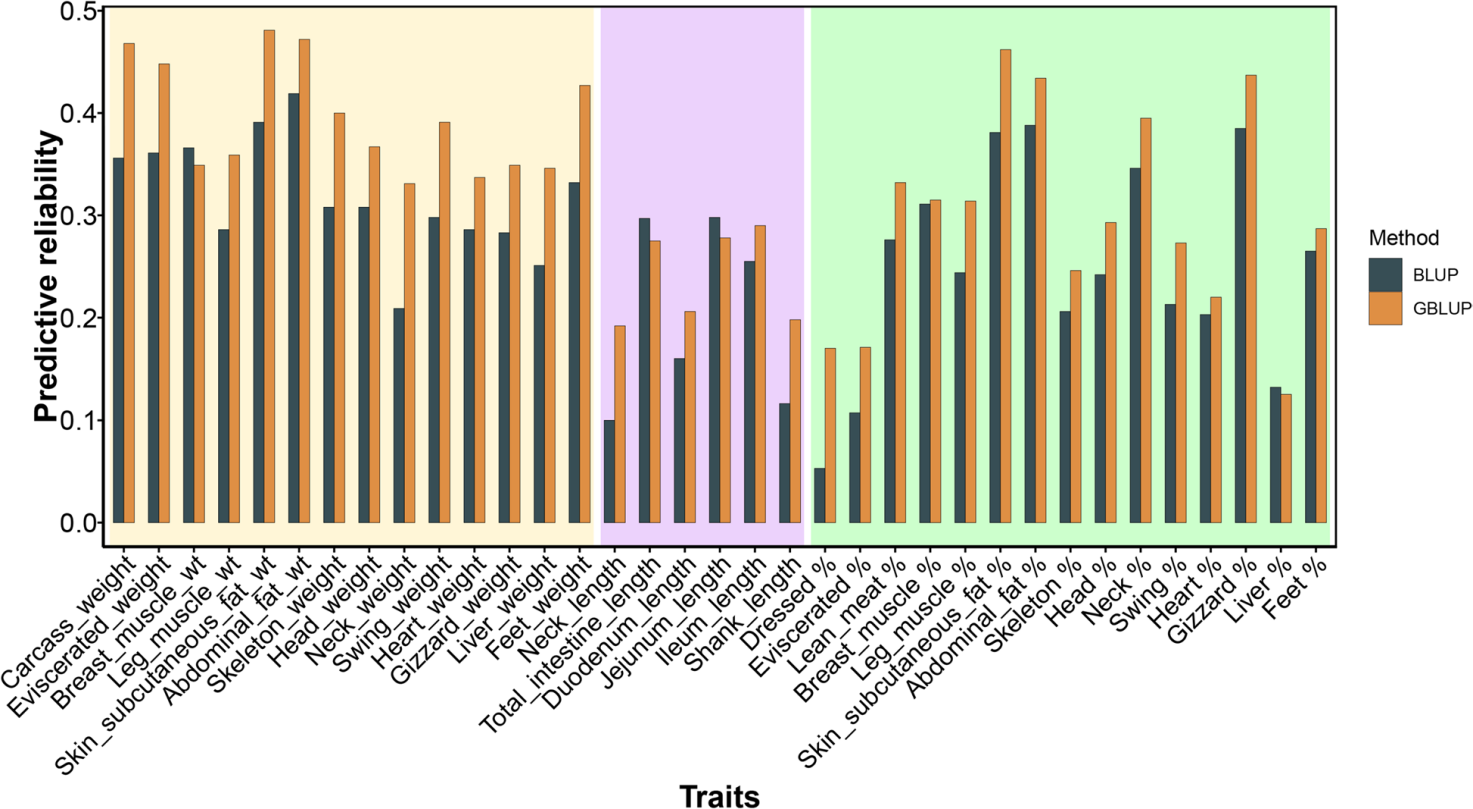
The predictive reliability of duck carcass traits by GBLUP and pedigree BLUP. The average of predictive reliability was calculated by 5-fold cross-validation. The predictive reliability of GBLUP and pedigree BLUP were denoted by the black and orange bars, respectively. The three colors in the background represent the different trait groups

The genomic predictive reliability for carcass traits using pedigree BLUP and GBLUP methods are summarized in Table S2 and Table 2. Globally, the reliability of genome prediction (GBLUP) varied from 0.12 to 0.48 for carcass traits (Fig. 2). The reliability of genome prediction was relatively high for several weight traits, such as skin and subcutaneous fat weight (0.48), abdominal fat weight (0.47), carcass weight (0.47), skin and subcutaneous fat percentage (0.46), eviscerated weight (0.45), gizzard percentage (0.44), while we observed low predictive reliability for liver percentage (0.13), dressed rate (0.17), eviscerated percentage (0.17), neck length (0.19) and shank length (0.20). Compare to the conventional pedigree BLUP strategy, the predictive ability of the GBLUP model was significantly higher (*P* < 0.001, paired *t*-test). For each trait, we observed 32 out of 35 traits were increased by genome prediction. The increment in predictive ability obtained using GBLUP respect to pedigree BLUP was more noticeable in the neck weight (0.12), dressing rate (0.12), and carcass weight (0.11). The average increment of predictive reliability between GBLUP and BLUP was 0.06 (ranged from −0.02 to 0.12) across all 35 traits.

### The marker density affects genome predictions

**Fig. 3:**
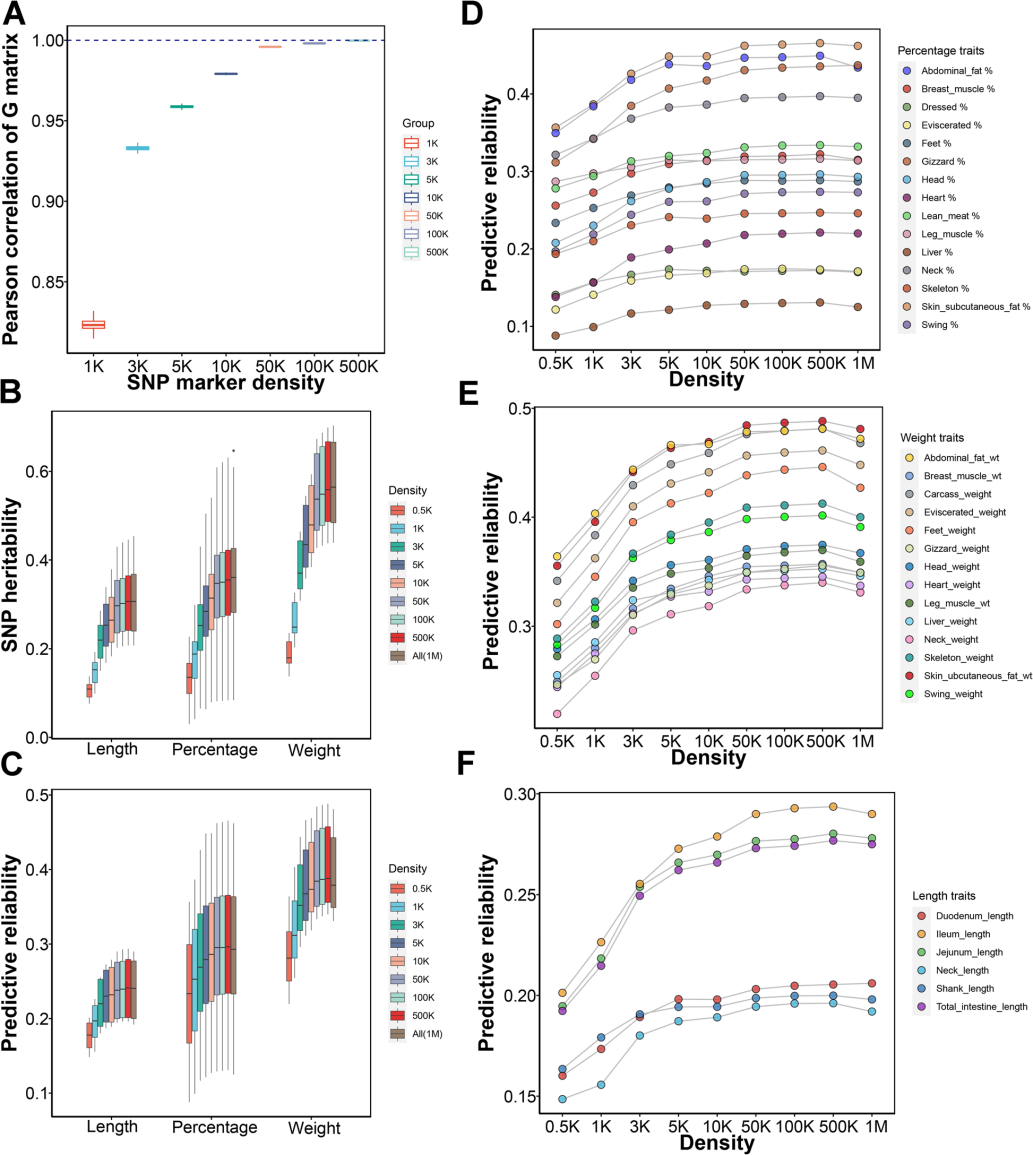
The permutation of marker density affects the estimation of SNP heritability and predictive reliability of GS in duck carcass traits. **A** Pearson correlation coefficients between all genomic relationship matrixes built from 30 times randomly selected markers. **B** The estimation of SNP heritability was increased with a high density of markers in three trait groups. All sequencing variants (1.04M) were also used to compute the SNP heritability, which was marked with the last brown color. **C** The predictive reliability of GS changes by the various markers’ density in three trait groups. **D–F** The predictive reliability of GS changes by the various markers density across each trait for percentage traits, weight traits and length traits

We randomly selected 0.5K, 1K, 3K, 5K, 10K, 50K, 100K, and 500K, from the original sequencing markers, each permutation was repeated 30 times. To check whether the marker density could affect the genomic relationships, we calculated the Pearson correlation coefficients between permutations in each density group after building the GRM. We found that the correlation coefficients between permutations rapidly increased from 0.5K to 3K density, and moderately increased from 3 to 50K density (Fig. 3A). The correlation coefficients tended to be steady with the average value exceeding 0.996 for 50K SNPs. We observed that estimated SNP heritabilities increased rapidly with the density increasing from 0.5K to 50K, and then slightly increased with 50K higher density (Fig. 3B). The SNP heritabilities of permutations were listed in Table S3. The increment of SNP heritabilities was more noticeable for weight traits with high heritabilities (Fig. S1). The predictive reliability for each trait was calculated by averaging the cross-validation results of 30 random permutations. We found that the predictive reliability rapidly increased with the increase of marker density from 0.5K to 3K, then moderately increased from 3 to 5K. The predictive reliability was limited improvement when the marker density exceeded 50K (Fig. 3C). The average of predictive reliability was 0.27 for 1K SNP markers, which is close to reliability of pedigree BLUP (Table S4). The predictive capability (current reliability divided by the best performance of reliability within the nine different density markers) of 50K density reached 99%. (Fig. S2 and Fig. 3D–F). The 3K SNP markers with a predictive capability of 90.7% needed attention (Fig. S2). It should be noticed that the predictive reliability of most traits would not be increased when we fitted all 1.03M sequence variants (Fig. 3D–F).

### The GRM methods affect the GBLUP performance

**Fig. 4:**
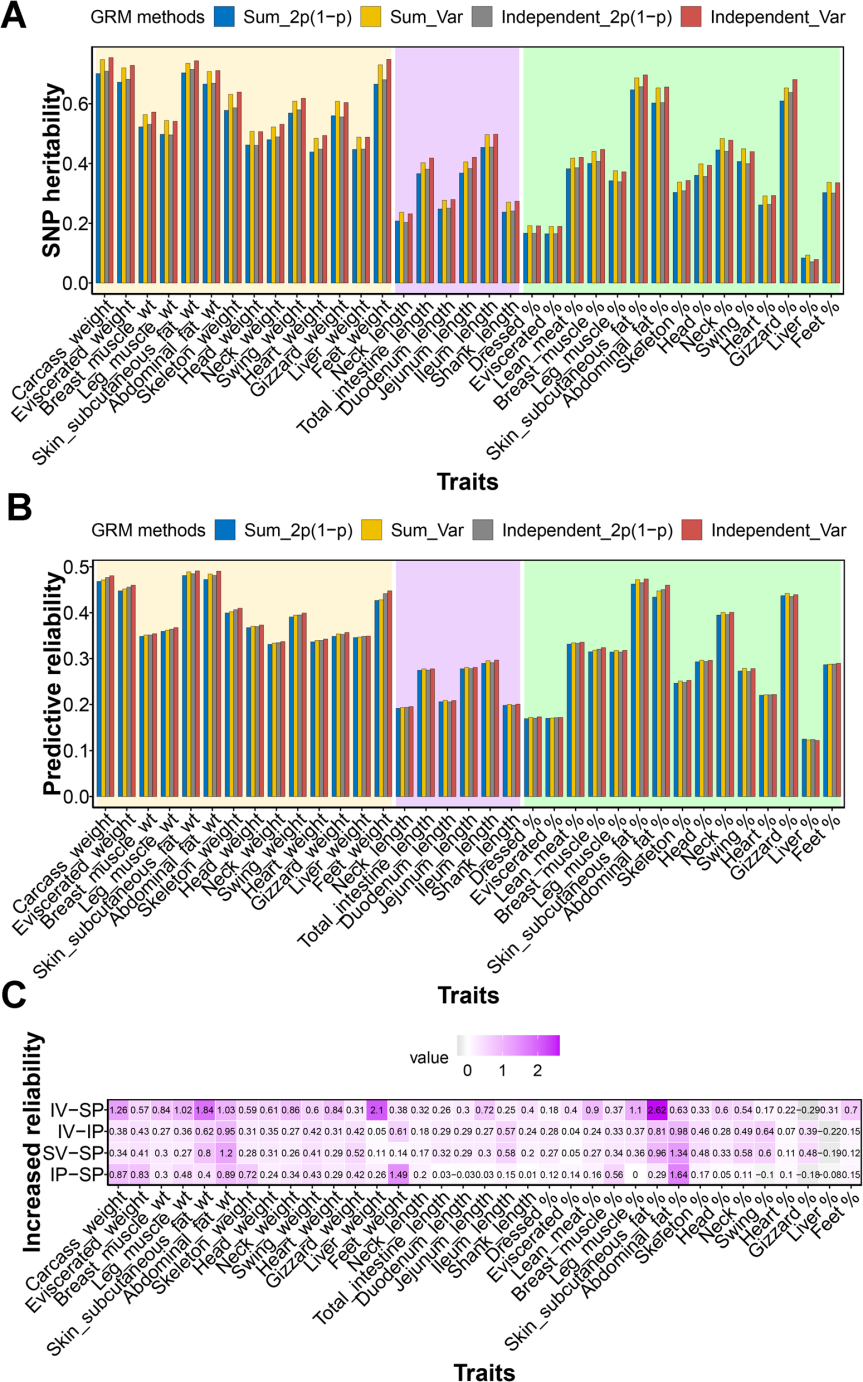
The predictive reliability of duck carcass traits by different GRM methods implemented in GBLUP model. **A** The SNP heritabilities were estimated by four GRM methods implemented in GBLUP for 35 duck carcass traits. **B** The predictive reliability of GBLUP with four GRM methods for carcass traits. **C** The increased gains between four GRM methods in predictive reliability of carcass traits. The label in each box is the percentage value of the gained reliability between the two methods. SP: sum 2*p*(1 − *p*), IP: Independent 2*p*(1 − *p*), SV: sum true variance, IV: Independent true variance. The IV-SP on *y*-axis means the predictive reliability gain in IV compared to SP

The genomic matrix methods proposed by VanRanden [16] and Yang et al. [17] was widely used in animal breeding and human genetics study. The difference between the two methods lies in correcting the genotype variance of ***ZZ****'.* VanRaden [16] believed that all SNPs should be corrected by an equal variance $\sum {2p}_{i}(1-{p}_{i})$, while Yang et al. [17] argued that each SNP should be independently corrected itself variance $2{p}_{i}\left(1-{p}_{i}\right)$. Here we found the independent $2{p}_{i}\left(1-{p}_{i}\right)$ method had a significantly better performance in both SNP heritability (paired *t*-test *P* < 2.29E−05) and GBLUP reliability (paired *t*-test *P* < 1.78E−05) than sum $2{p}_{i}\left(1-{p}_{i}\right)$ in most carcass traits (Fig. 4A and B). The increment in SNP heritability and predictive ability obtained using independent $2{p}_{i}\left(1-{p}_{i}\right)$ respect to sum $2{p}_{i}\left(1-{p}_{i}\right)$ was 0.0041 and 0.0033, respectively (Table S5). Then we proposed to use the ${p}_{1i}$ and ${p}_{2i}$ for the frequencies of genotype 1 and 2 instead of MAF ${p}_{i}$, which calculated the true variance of genotype for each SNP was ${[p}_{1i}+4{p}_{2i}-{{(p}_{1i}+{2p}_{2i})}^{2}$] rather than $2{p}_{i}\left(1-{p}_{i}\right)$. Compared to GRM using the experienced $2{p}_{i}\left(1-{p}_{i}\right)$ method, using our true variance method on both two methods could capture more SNP heritability (paired *t*-test *P* < 4.16E−23 for sum variance, and *P* < 2.72E−22 for independent variance). The average of SNP heritability gained was 0.039 for both sum and independent true variance methods. For the prediction, we also found a significant improvement in predictive reliability of GS for most traits in both sum (paired *t*-test *P* < 4.77E−9) and independent (paired *t*-test *P* < 7.86E−11) methods by true variance (Fig. 4C). Compare with the widely used VanRaden's method, using the independent true variance of GRM could obtain an average of 0.007 increments in reliability of all traits (Table 2 and Table S5). The reliability increment obtained using the independent true variance method was more noticeable in these traits with high heritabilities, such as the abdominal fat percentage (0.026), feet weight (0.021), head weight (0.019), abdominal fat weight (0.019), and carcass weight (0.013) and eviscerated weight (0.013).

### Bayesian models can improve the prediction accuracy

**Fig. 5:**
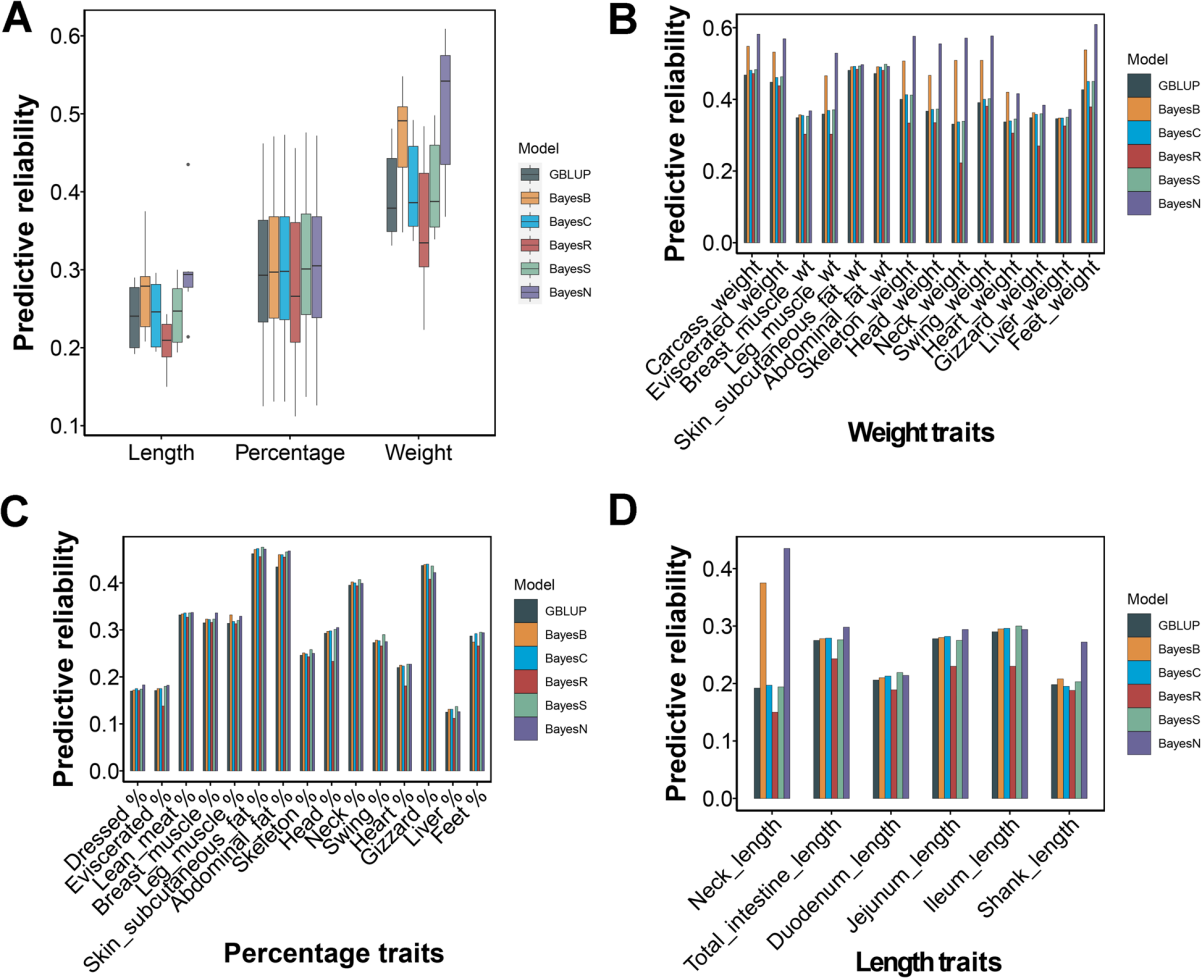
The predictive reliability of duck carcass traits by Bayesian models. **A** The predictive reliability of GS changed using GBLUP and different Bayesian models in three trait groups. **B–D** The predictive reliability of GS varied using GBLUP and different Bayesian models in each trait for weight traits, percentage traits and length traits

The reliability of genome prediction was greater for the Bayesian models (except for BayesR) than for the GBLUP models in most traits, while the advantage of Bayesian models in percentage traits was meager (Fig. 5A). For each Bayesian model, both BayesN and BayesB achieved visible performance for most weight traits and length traits (Fig. 5C–D). The BayesN was the best model for increasing the predictive reliability of most carcass traits (Fig. B–D and Table 2). The increment in predictive ability obtained using BayesN respect to GBLUP was more noticeable in the neck length (0.24), neck weight (0.24), head weight (0.19), swing weight (0.19), and feet weight (0.18) (Table S6). To our surprise, we found BayesR had a poor performance in most traits, even worse than GBLUP. Interestingly, the BayesS model, accounting for MAF and LD weights of markers, had the best reliability performance in several percentage traits, such as skin and subcutaneous fat percentage, skeleton percentage, neck percentage, swing percentage, liver percentage and feet percentage (Table 2 and Table S6). The reliability of genome prediction using BayesN increased by an average of 0.117 compared to pedigree BLUP.

**Table 2:** The predictive reliability of (genomic) breeding values for duck carcass traits using different strategies

Methods	Weight traits	Length traits	Percentage traits	All 35 traits
BLUP	0.318	0.205	0.250	0.269
Sum *2p*(1 − *p*) GBLUP	0.395	0.240	0.298	0.327
Independent 2*p*(1 − *p*) GBLUP	0.400	0.240	0.300	0.330
*Sum true variance GBLUP	0.399	0.243	0.302	0.331
*Independent true variance GBLUP	0.404	0.243	0.304	0.334
BayesB	0.468	0.274	0.304	0.364
BayesCπ	0.405	0.244	0.305	0.334
BayesR	0.360	0.205	0.285	0.301
BayesS	0.407	0.244	0.308	0.337
BayesN	0.507	0.301	0.307	0.386

Data showing the average of predictive reliability in each trait group. The predictive reliability is computed using 5-fold cross-validation. The true variance method, denoted with an asterisk (*), is a newly proposed method for improving GRM in the GBLUP model

## Discussion

Genomic selection expects to speed up genetic progress in animal breeding programs [35]. Practically, to implement GS in duck breeding, it is necessary to know the performance of GS in predicting GEBV, as well as assess various marker densities and GS models to create appropriate strategies for an effective breeding program in ducks. In this study, we estimated the genetic parameters, performed GS using different models and marker densities, and compared the estimation performance between GS and traditional BLUP on 35 carcass traits in an F_2_ population of ducks.

### Genetic parameters of 35 carcass traits

There are few reports on the genetic parameters of duck carcass traits [4, 36–38]. We provide the most comprehensive estimates of genetic parameters for carcass traits to date. Most of the 35 traits were never reported before. Most weight traits were estimated to be high heritabilities (> 0.4), except for neck and liver weight with moderate heritabilities. The high heritabilities in weight of carcass, wing, breast muscle, leg muscle, skin and subcutaneous fat, abdominal fat and skeleton were also reported in another F_2_ crossbreed of Pekin type ducks study (0.47–0.75) [36]. Moderate heritability was obtained for the liver weight (0.32), which was similar to the results of 0.29 by Mucha et al. [36] and 0.36 by Deng et al. [38]. This is the first study to report the heritability of heart (0.41) and gizzard (0.41) weight in ducks, which were close to broiler chicken’s study (heart weight: 0.41 and gizzard weight: 0.41) [39]. The duck products of the intestine and neck were popular in Asia countries. However, the length heritabilities of the intestine, neck and shank have not been reported before. Here we observed that intestine length traits were moderate heritabilities, while the length of the shank and neck were low heritabilities. The genetic mechanisms of weight percentage traits were complicated, which were calculated by dividing the two traits, resulting in dynamic heritabilities. The breast muscle percentage (0.38) was lower than the result (0.47) shown by Xu et al. [37], but higher than the result (0.16) shown by Xu et al. [4]. The leg muscle percentage (0.30) and abdominal fat percentage (0.56) were higher than the result (0.16 and 0.32, respectively) shown by Xu et al. [37].

Both phenotypic and genetic correlations of weight traits were usually positive and high. Similar results for duck populations were generally reported by previous studies [36, 37, 40, 41]. The strongest correlations between jejunum length and total intestine length were in a greement with the biological background of particular recorded traits. In the study by Mazanowski et al. [41], breast and leg muscle weight, and carcass weight positively correlated with shank length and trunk with neck length, which was partly confirmed in our study. Dressing percentage showed positive and low correlations with weight traits, which is consistent with a previous study [40]. Some percentage traits with large gaps between genetic and phenotype correlation implied the complicated genetic mechanisms in these ratio-recorded traits.

### Genome prediction performance

The high predictive ability of GS in skin and subcutaneous fat weight, abdominal fat weight, carcass weight, skin and subcutaneous fat percentage, eviscerated weight, and gizzard percentage, suggests that the better performance of GS could be found in traits with high heritability. Similar results were seen in other research where there was a significant association between trait heritability and prediction reliability [42, 43]. This phenomenon was reported in other species. The skin and subcutaneous fat had a relatively high prediction ability, which was also observed in Pekin ducks of a previous study [9]. We found an obvious benefit of GS in predicting the breeding values. The reliability of genome prediction using GBLUP increased by an average of 0.06 compared to BLUP. These improvements are consistent with validation results of GS in other poultry [7, 44] or livestock [6, 45, 46]. Among the 35 traits in this study, using GS improved reliabilities most for neck weight and dressing rate, which indicated GS might have more potential in low heritabilities.

In Bayesian models, the differences among methods are the assumptions on the genetic marker effects, which outperform GBLUP when the number of quantitative trait loci (QTLs) underlying the trait is smaller than the number of independent chromosome segments [47]. In this study, we found most of the Bayesian models had a better performance than the GBLUP method, which implied these carcass traits are controlled by a limited number of major QTLs. We have found that the BayesN method was the most accurate method to predict breeding values in most traits. The advantage of BayesN related method was also reported by Zeng et al. [48] and Karaman et al. [49]. BayesR had a poor performance in the carcass traits of our population. BayesR could not increase the accuracy of genomic prediction compared to GBLUP in other studies [50, 51]. BayesR model assumpted four marker distributions, can shrink large effects heavily, which tend to overperform GBLUP when a small number of loci with large effects exist in trait [52]. Such major loci may be rare in carcass traits of this study. The reliability of genome prediction using BayesN could bring an average increase of 0.117 compared to pedigree BLUP, which further verified that the GS is promising in duck breeding programs.

### Effects of marker density on GS

An increase in marker densities generally resulted in raised accuracy predicted. In our study, the marker density of 1K could achieve the predictive ability of the traditional BLUP breeding strategy, which indicated GS has great potential in broiler duck breeding. The predictive ability was dramatically increased when marker density was below 3K, then the increase of predictive ability was slowed down. With a marker density of 50K, the prediction accuracy for most carcass traits reached a plateau. A similar phenomenon was found in other species although the threshold might be different [13, 53]. The threshold of the plateau might be affected by the linkage disequilibrium of markers. The number of independent segments is usually small in populations with strong LD, which means fewer markers are needed to capture all segments [54]. The 50K marker density could achieve 99% of predictive capability, which suggesting 50K density marker can achieve ideal predictive ability for duck carcass traits. The 3K marker density still had a high predictive capability (90.7%), which implied that it is feasible to reduce breeding costs by designing low-density chips on ducks.

### The improvement of GS by modifying GRM

In recent years, GBLUP has been a widely used method for genomic evaluation in livestock. The predictive ability of genomic breeding values estimated by GBLUP can be affected by the characteristics of GRM, which was significantly affected by the number of markers, markers’ weights and standardized methods [16, 52]. Although VanRaden [16] suggested ***ZZ'*** should be corrected by $\sum {2p}_{i}(1-{p}_{i})$ in dairy cattle breeding, we found both SNP heritabilities and GS reliability were improved by the standardized method of independent $2{p}_{i}\left(1-{p}_{i}\right)$ of each SNP in duck carcass traits. We guess that small animals may be more often selected than large livestock, which causes the genotype variance of each SNP to not be equal. We proposed the true variance method using the ${p}_{1i}$ and ${p}_{2i}$ for the frequencies of genotypes 1 and 2 instead of MAF ${p}_{i}$. Then we found that the true variance method robustly achieved high performance in both SNP heritability and GS reliability. When the population obeys the Hardy–Weinberg principle, the genotype variance of each SNP was equal to $2{p}_{i}\left(1-{p}_{i}\right)$ (See Method). However, in most cases, the SNPs were not fully satisfied for Hardy–Weinberg equilibrium, especially for a small selected population, which lead to the bias of variance evaluation using $2{p}_{i}\left(1-{p}_{i}\right)$, which may explain the reason for the improvement in both SNP heritability and GS reliability using our true variance method. The independent true variance method of GRM could bring more noticeable improvement in both SNP heritability and GS reliability in most traits, which was worth further investigation and use in other species.

## Conclusions

Our results demonstrate that genomic prediction is a feasible approach for accurate selection in duck breeding programs, especially for these traits which are difficult to be measured such as carcass traits. The genomic prediction can be further improved by modifying the GRM using our true variance method, which is worth promoting in GS. Several Bayesian models, especially for BayesN, could bring more noticeable improvement in the predictive ability of GS, which need attention. The permutation studies of density markers indicate 50K markers achieved ideal prediction accuracy, while 3K markers still achieved 90.7% predictive capability, which would be promised to further reduce cost in duck breeding. In conclusion, our findings offer some useful strategies for the optimizing predictive ability of GS and provide theoretical foundations for designing a low-density panel in ducks.

## Supplementary Information

**Additional file 1: Table S1.** Estimation of phenotypic correlation and genetic correlation for the 35 carcass traits.

**Additional file 2: Table S2.** Estimation of SNP heritabilities for duck carcass traits using different marker densities.

**Additional file 3: Table S3.** The predictive reliability of genomic breeding values for duck carcass traits using different marker densities by GBLUP model.

**Additional file 4: Table S4.** The SNP heritabilities and prednishiictive reliability of genomic breeding values for duck carcass traits using four different GRM methods.

**Additional file 5: Table S5.** The predictive reliability of genomic breeding values for duck carcass traits using Bayesian models.

**Additional file 6.**

**Additional file 7: Fig. S1.** The permutation of marker density affects the estimation of SNP heritability of GS in duck carcass traits. (A–C) The SNP heritability changes by the various markers' density across each trait for weight traits (A), length traits (B) and percentage traits (C).

**Additional file 8: Fig. S2.** The predictive capability of genomic breeding values for duck carcass traits using different marker densities. The color of each box represents a high capability (red) or a low capability (blue).

## Data Availability

All the gennotype data have been deposited in the Sequence Read Archive (https://www.ncbi.nlm.nih.gov/sra) with the accession numbers PRJNA471401 and PRJNA450892.

## Abbreviations

BLUP: Best linear unbiased prediction
GBLUP: Genomic best linear unbiased prediction
GRM: Genomic relationship matrix
GS: Genomic selection
IP: Independent 2*p*(1 − *p*)
IV: Independent ture variance
MAF: Minor allele frequency
MCMC: Markov chain Monte Carlo
REML: Restricted maximum likelihood
SD: Standard deviation
SNP: Single nucleotide polymorphism
SP: Sum 2*p*(1 − *p*)
SV: Sum ture variance
