# Supplementary figures and images for: Strategies to improve genomic predictions for 35 duck carcass traits in an F2 population
Source: J Anim Sci Biotechnol. 2023 May 6;14:74. doi: 10.1186/s40104-023-00875-8 (PMC10163724; doi:10.1186/s40104-023-00875-8)

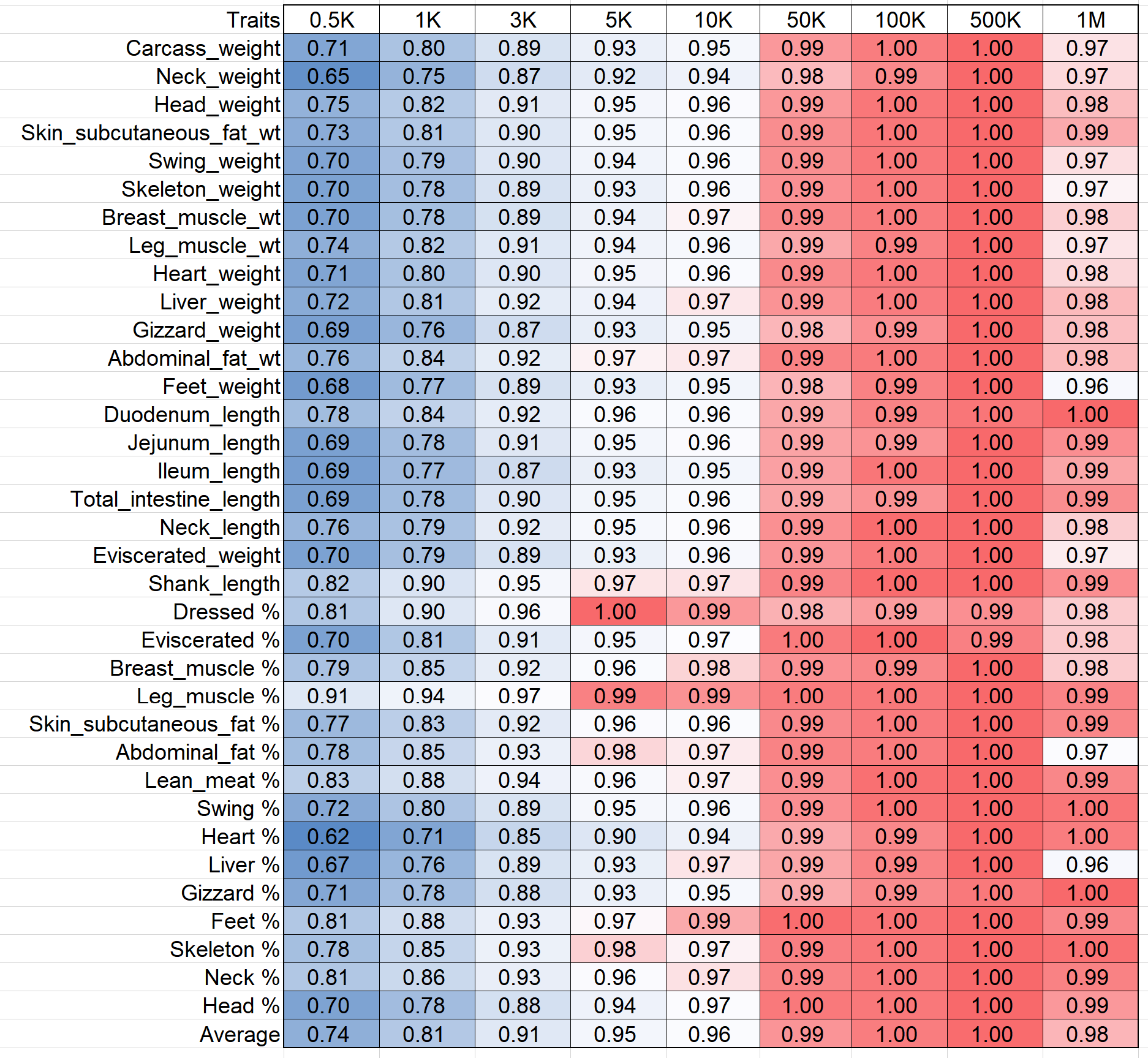

Supplement: Supplementary file 7 — Additional file 7: Fig. S1. The permutation of marker density affects the estimation of SNP heritability of GS in duck carcass traits. (A–C) The SNP heritability changes by the various markers' density across each trait for weight traits (A), length traits (B) and percentage traits (C). [file 40104_2023_875_MOESM7_ESM.tif]

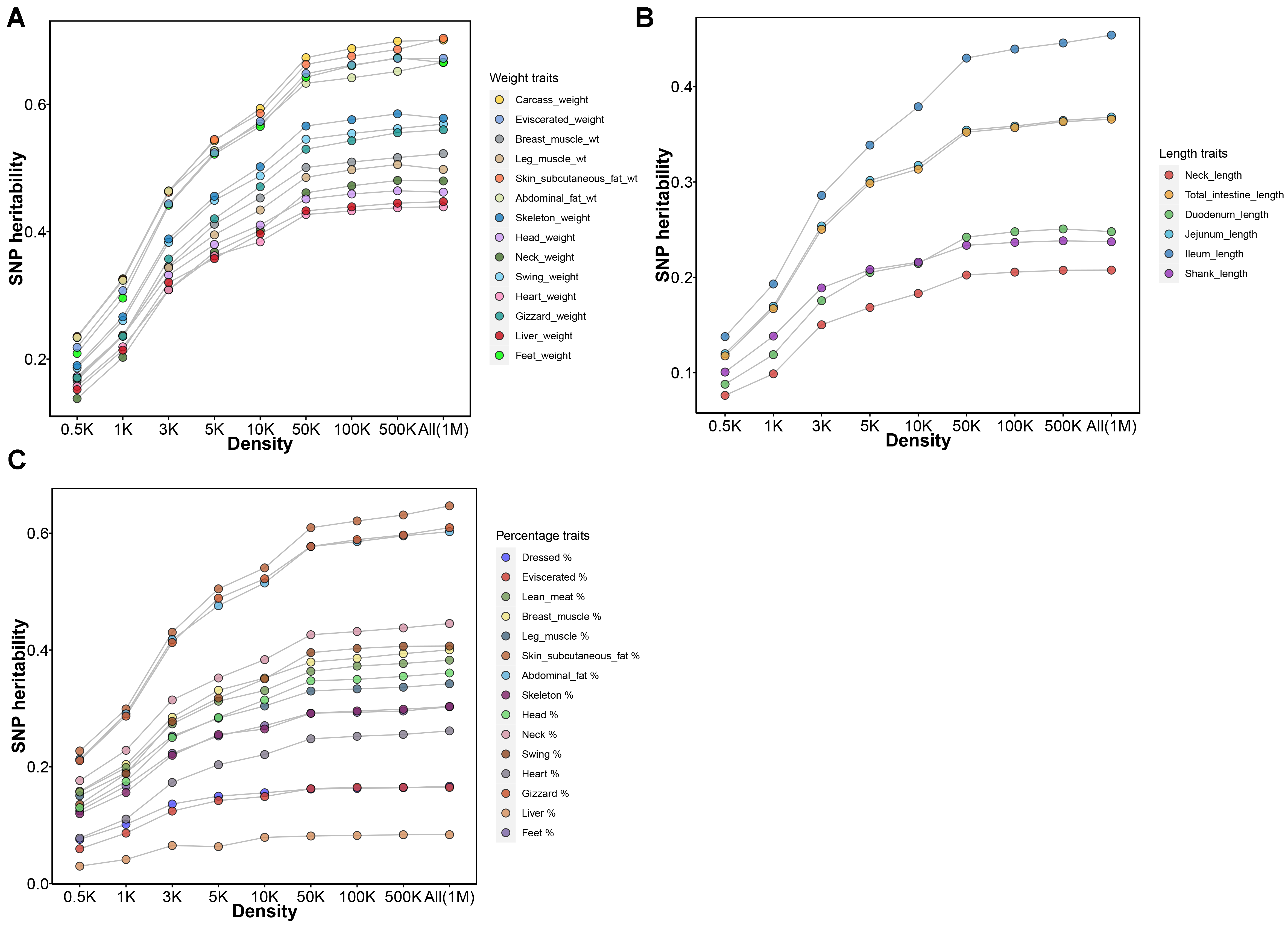

Supplement: Supplementary file 8 — Additional file 8: Fig. S2. The predictive capability of genomic breeding values for duck carcass traits using different marker densities. The color of each box represents a high capability (red) or a low capability (blue). [file 40104_2023_875_MOESM8_ESM.tif]
